# Nivolumab induced encephalopathy in a man with metastatic renal cell cancer: a case report

**DOI:** 10.1186/s13256-018-1786-9

**Published:** 2018-09-15

**Authors:** Jindřich Kopecký, Ondřej Kubeček, Tomáš Geryk, Birgita Slováčková, Petr Hoffmann, Miroslav Žiaran, Peter Priester

**Affiliations:** 1 Department of Clinical Oncology and Radiotherapy, University Hospital in Hradec Králové, Sokolská 581, 50005 Hradec Králové, Czech Republic; 2 Department of Fingerland Pathology, University Hospital in Hradec Králové, Sokolská 581, 50005 Hradec Králové, Czech Republic; 3 Department of Psychiatry, University Hospital in Hradec Králové, Sokolská 581, 50005 Hradec Králové, Czech Republic; 4 Department of Radiology, University Hospital in Hradec Králové, Sokolská 581, 50005 Hradec Králové, Czech Republic

**Keywords:** Renal cancer, Nivolumab, Encephalitis, Immune-related adverse event, Case report

## Abstract

**Background:**

Great progress has recently been made in the treatment of metastatic renal cell carcinoma, including the introduction of nivolumab, an immune checkpoint inhibitor. Despite promising results, this treatment brings a completely new spectrum of adverse events, distinct from those experienced with small-molecule kinase inhibitors. Neurologic immune-related adverse events may be serious and potentially life-threatening complications requiring immediate immunosuppressive therapy. Only a few cases of immune-related encephalitis induced by checkpoint inhibitors have been described and the data regarding the management of this serious adverse event are limited.

**Case presentation:**

We report the case of a 63-year-old white man with metastatic renal cancer who developed severe chorea-like dyskinesia during nivolumab therapy. The findings on brain magnetic resonance imaging and flow cytometry of cerebrospinal fluid, and the positivity of anti-paraneoplastic antigen Ma2 immunoglobuline G class autoantibodies were consistent with a diagnosis of immune-related encephalitis. High-dose intravenous corticosteroid therapy was started immediately, with no signs of improvement, even when infliximab was added. Our patient refused further hospitalization and was discharged. Three weeks later, he presented with signs of severe urosepsis. Despite intensive treatment, he died 4 days after admission.

**Conclusions:**

The management of less frequent immune-related adverse events has not been fully established and more information is required to provide uniform recommendations. Immune-related encephalitis is a severe and potentially fatal complication requiring immediate hospital admission and extensive immunosuppressive therapy. The examination of cerebrospinal fluid for paraneoplastic antibodies, such as anti-N-methyl-D-aspartate receptor and anti-Ma2 antibodies, in order to distinguish autoimmune etiology from other possible causes is essential and highly recommended.

**Electronic supplementary material:**

The online version of this article (https://doi.org/10.1186/s13256-018-1786-9) contains supplementary material, which is available to authorized users.

## Background

Great progress has recently been made in the treatment of metastatic renal cell carcinoma (mRCC). Currently available drugs include multikinase vascular endothelial growth factor (VEGF) inhibitors (sunitinib, sorafenib, pazopanib), cytokines (interferon α), and mammalian target of rapamycin (mTOR) inhibitors (temsirolimus, everolimus), with the recent additions of the MEK inhibitor cabozantinib and the immune checkpoint inhibitor nivolumab. Nivolumab is a fully human immunoglobuline (Ig) G4 antibody targeting programmed cell death-1 (PD-1) receptor, which achieves a durable objective response in many cancers including mRCC [1]. Nivolumab acts as a checkpoint inhibitor, preventing the PD-1 mediated transmission of inhibitory signals that would normally attenuate T cell activity. This consequently enables the immune system to regain or maintain its antitumor activity. The anti-PD-1 effect is achieved mainly in tumor tissue during the effector phase of the immune response. Nivolumab is administered intravenously at a dose of 3 mg/kg every 14 days.

The advent of immunotherapy with checkpoint inhibitors has resulted in a completely different spectrum of activity than that experienced with chemotherapy and small-molecule kinase inhibitors. Desired antitumor activity can be achieved in a considerable number of patients. However, stimulation of the immune system response may simultaneously induce symptoms resembling an autoimmune disorder. These adverse reactions are usually referred to as an immune-related adverse event (irAE) and may affect practically any organ or tissue in the human body. Although these adverse reactions are usually mild and easily manageable with appropriate treatment, severe complications with potentially fatal consequences may occur.

We report a case of a patient with mRCC who developed severe chorea-like dyskinesia during therapy with nivolumab. The aim of this case report was to present a rare neurological complication of nivolumab treatment and to emphasize the necessity of close collaboration among the physician, the patient, and the patient’s family as a prerequisite for a good clinical outcome.

## Case presentation

**Fig. 1:**
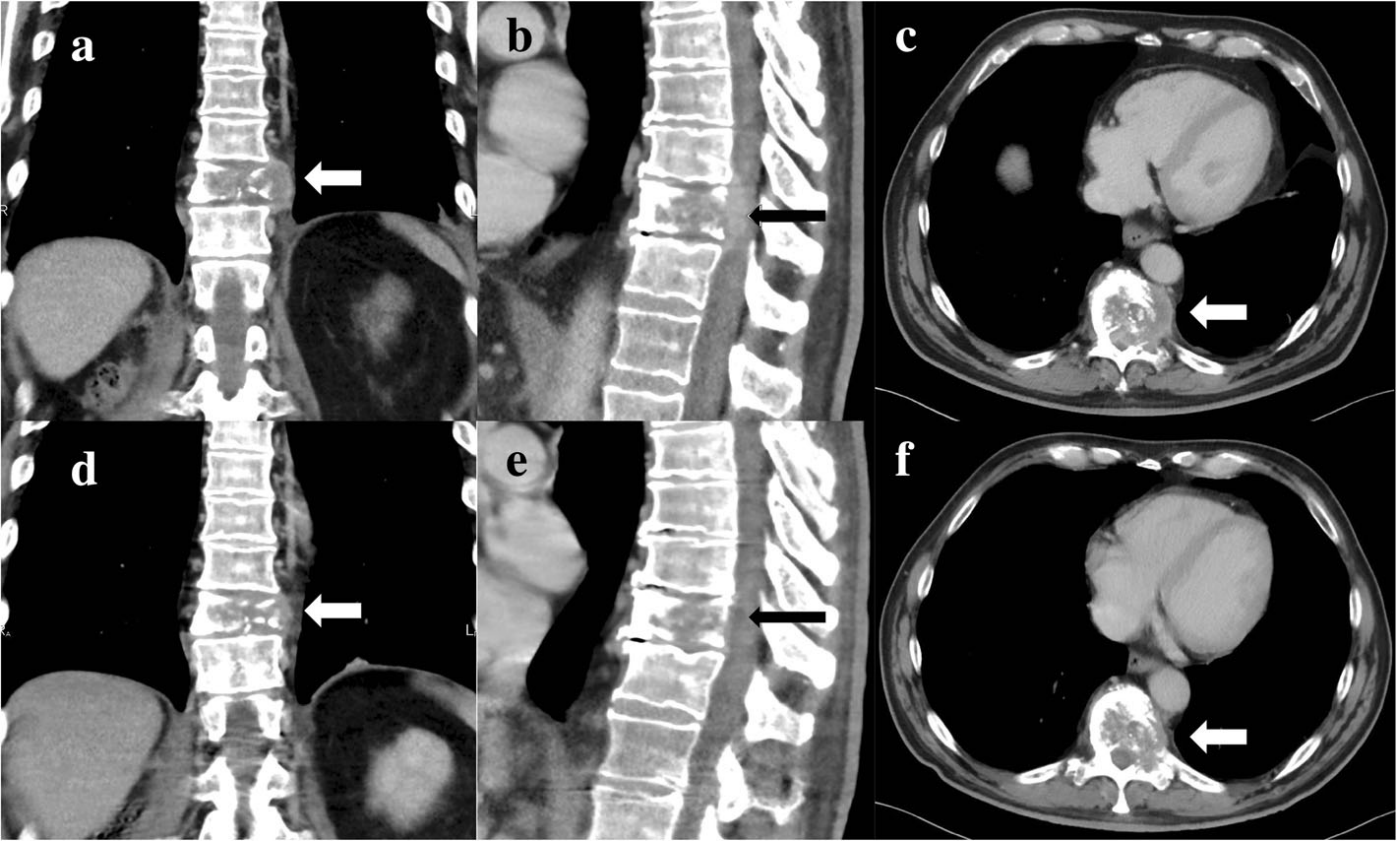
Frontal, sagittal, and axial computed tomography scan demonstrating a destructive mass affecting Th11body (see arrows) from April 2016 (**a**–**c**) and August 2016 (**d**–**f**)

**Fig. 2:**
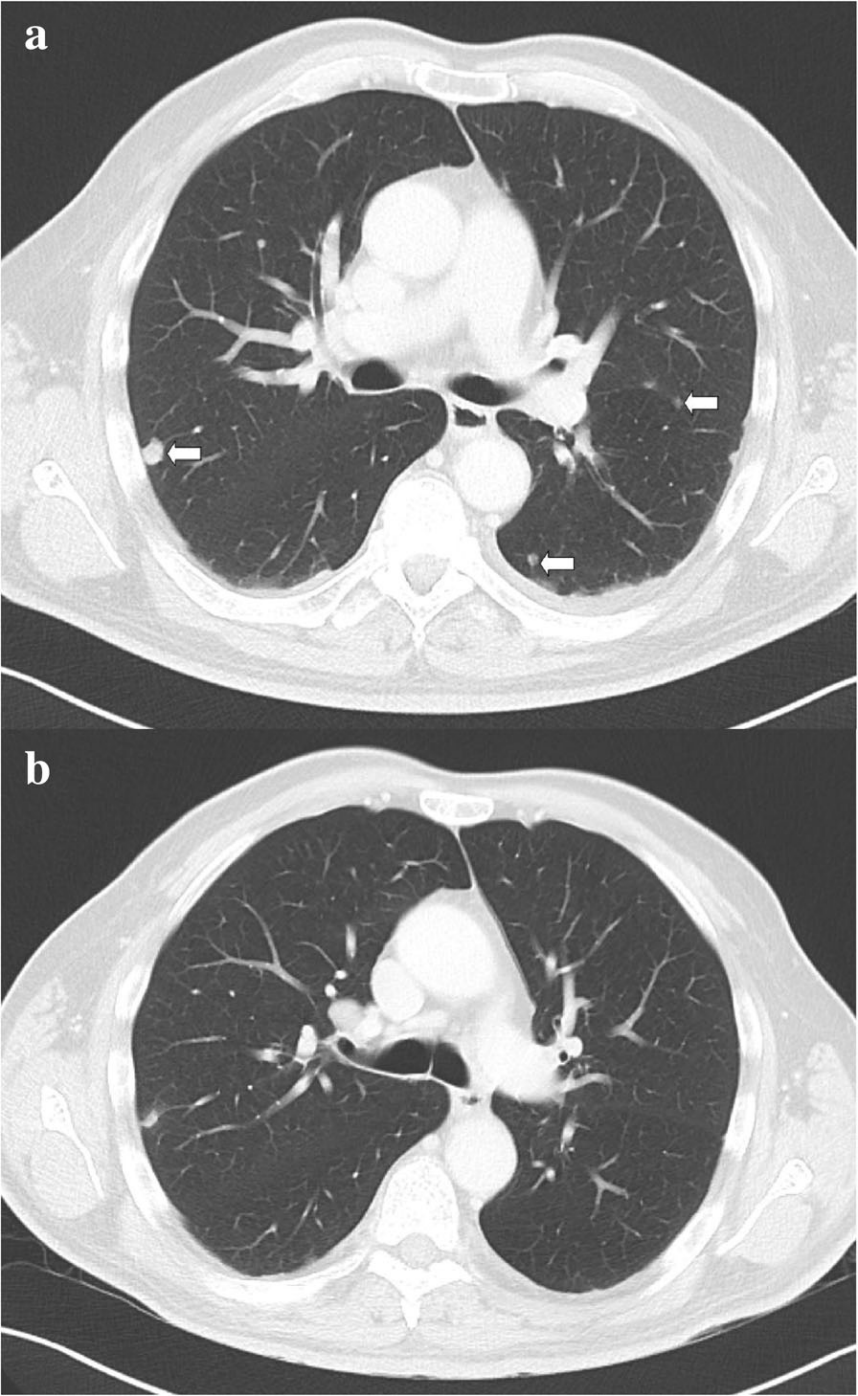
Axial contrast-enhanced computed tomography scans of the thorax showing tumor regression (see arrows) April 2016 (**a**) and August 2016 (**b**)

A 63-year-old white man with no significant comorbidities was diagnosed as having mRCC affecting his right kidney with metastatic spread in the Th11 vertebra and multiple pulmonary sites (Figs. 1a–c, 2a). He underwent a cytoreductive nephrectomy in December 2015. A histological examination was consistent with clear cell carcinoma, predominantly grade 2–3 (focally grade 4) with small areas of sarcomatoid differentiation and necrosis. The tumor stage was assessed as pT1b pN1 cM1. He was sent to the Comprehensive Cancer Center of the University Hospital in Hradec Králové, where he started therapy with sunitinib (50 mg daily, 4 weeks on/2 weeks off schedule) in December 2015. Considering the bone metastases, treatment with denosumab was started simultaneously. Owing to poor tolerability (nausea, fatigue, and anorexia) of the treatment, the schedule was changed to 2 weeks on/1 week off. Due to progressive back pain, combined analgesic therapy with opiates was required (oxycodone, transdermal fentanyl patches). Disease progression was documented in his lungs and spine after 4 months on sunitinib in April 2016. His progressive back pain resulted in hospital admission to perform analgesic radiotherapy to the Th 9–12 area with a dose of 20 Gy in five fractions on 5 consecutive days. He developed diarrhea during the hospitalization. A possible infectious etiology was ruled out with microbiological stool examination, as well as examination for *Clostridium difficile* and its toxin, and he was started on symptomatic therapy with an antidiarrheal treatment (diphenoxylate hydrochloride 2.5 mg three times a day) and probiotics.

After finishing radiotherapy, nivolumab therapy was started in May 2016 within an expanded access program at an absolute dose of 300 mg every 14 days. Both diarrhea and back pain were gradually resolving during treatment, enabling dose reduction of the opiates. Our patient completed a total of six doses of nivolumab with no laboratory or clinical signs of adverse effects.

However, 14 days following the last dose of nivolumab, he reported a change in behavior and a history of uncontrollable movements. His family started to say that he was strange and restless. He personally felt very well when taking nivolumab and the pain was even improving. He was fully aware of the uncontrollable movements, and although he could think rationally, he was not able to influence or stop them.

There was no family history of neurological or mental illness, and he denied any head trauma or neurological disorders in the past. A physical neurological examination revealed no significant findings in his head and peripheral nerves, but there were mild generalized choreatic movements of his upper extremities and head. A psychiatrist described our patient as cooperative, with pronounced choreatic movements of the entire body. His behavior was described as social, without signs of hostility or aggression, and at a reasonable psychomotor tempo. His mood was described as mildly dysphoric in response to the current situation of somatic manifestations. Laboratory tests showed no marked abnormalities. The only medication he was on at that time was a transdermal fentanyl patch (100 mcg/hour changed every 3 days), and he intermittently used antidiarrheal medications (diphenoxylate hydrochloride 2.5 mg or probiotics based on *Lactobacillus acidophilus* metabolites); during the sunitinib treatment, he irregularly used metoclopramide 10 mg, but he denied any history of neuroleptic use. Because of a serious suspicion of a possible side effect associated with immunotherapy, he was admitted to our hospital on 11 August 2016. A general overview of the timeline of the case report is shown in an additional file (see Additional file 1).

**Fig. 3:**
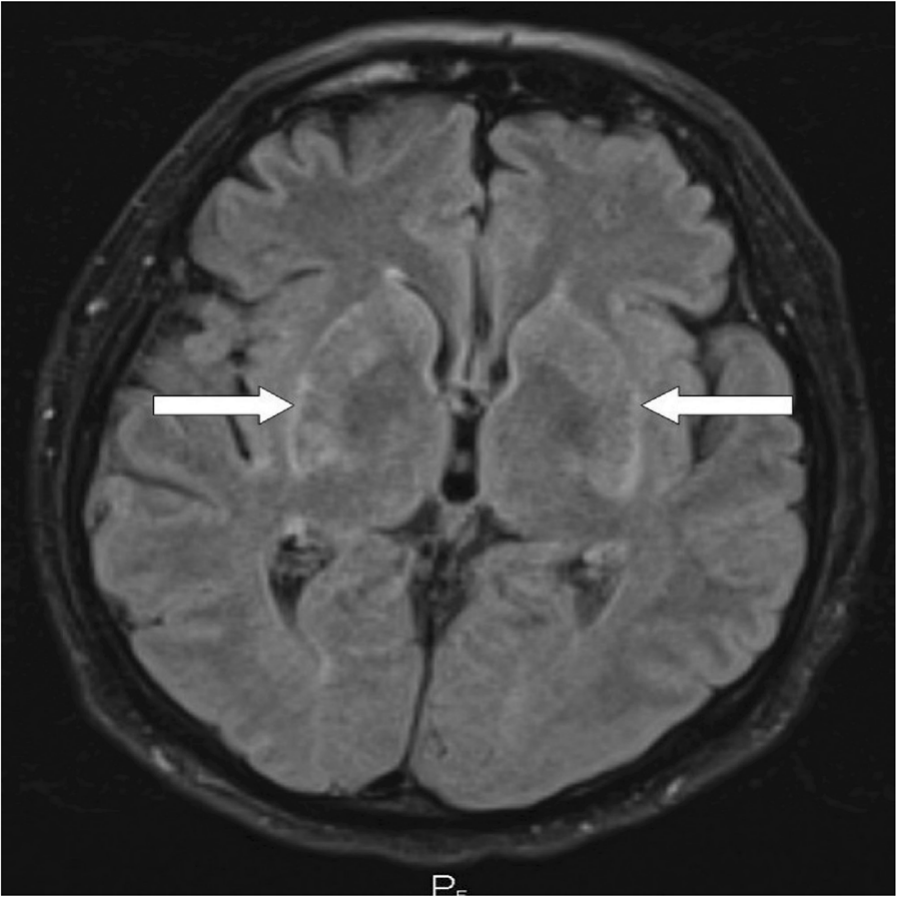
Susceptibility-weighted imaging magnetic resonance imaging of the brain showing (see arrows) an areaof inflammatory increased signal within the basal ganglia (August 2016)

CT (computed tomography) of his chest, abdomen, and pelvis showed signs of tumor regression in his lungs and bones (Figs. 1d–f, 2b). CT of his brain ruled out brain lesions or infiltrative brain damage. Because of the deterioration of choreatic movements, a magnetic resonance imaging (MRI) of his brain was performed. There were no signs of any tumor lesion. However, the MRI revealed a symmetrical, pathologically increased signal within the basal ganglia consistent with possible inflammatory involvement of these structures (Fig. 3).

**Fig. 4:**
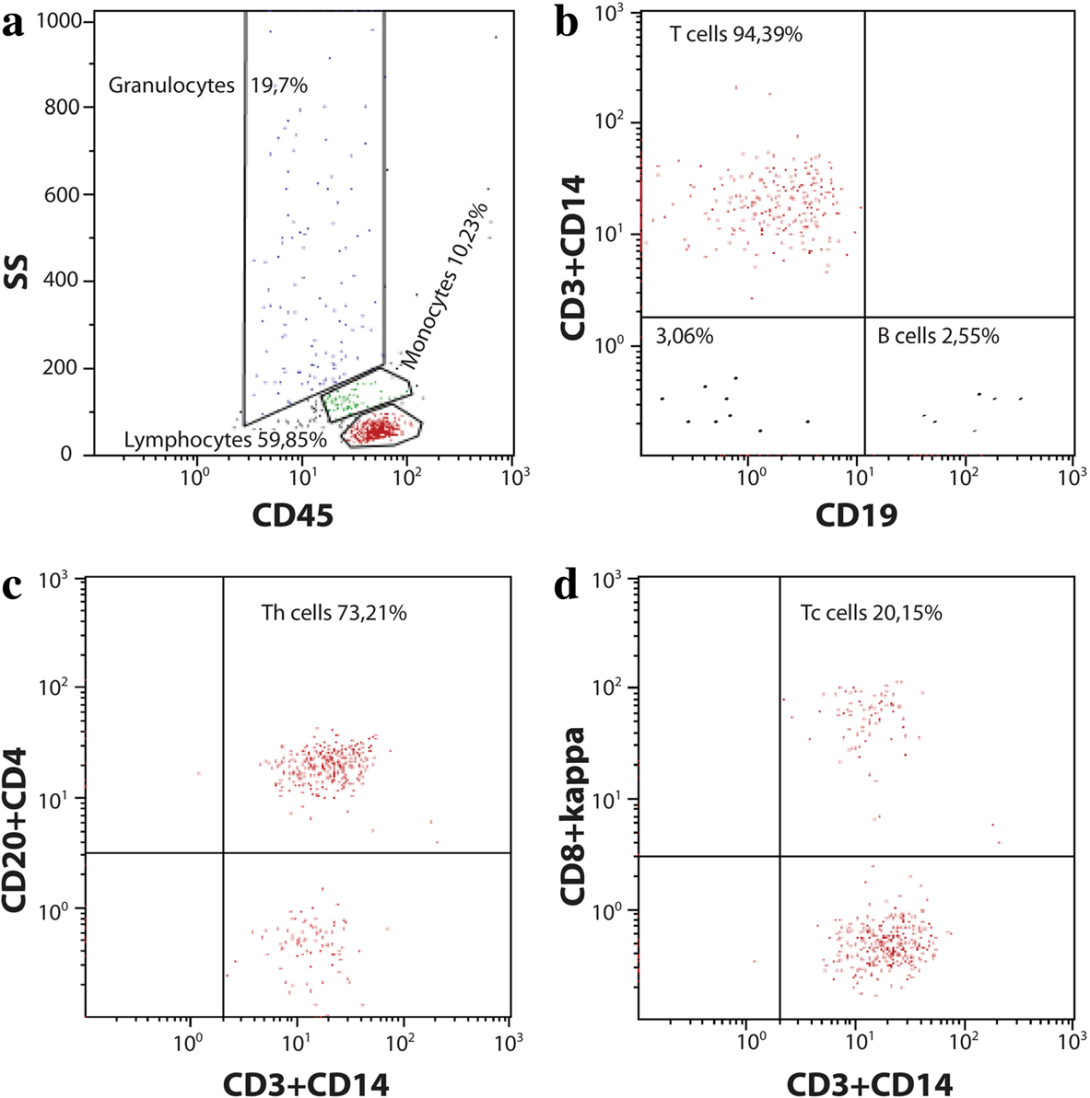
Flow cytometry gating of cerebrospinal fluid. **a** Side scatter versus CD45 plot for identification of basic population of leucocytes. **b** Identification of T (CD3+) and B (CD19+) cells. **c** Identification of Th (helper) cells (CD4+) and **d** Tc (cytotoxic) cells (CD8+). *SS* side scatter

Serum laboratory tests for infection and autoimmune diseases were negative. A cerebrospinal fluid (CSF) examination yielded negative results for bacterial and viral involvement and for the presence of malignant cells. Specific neuroimmunological examination of the CSF showed only mild inflammatory changes without any evidence of tissue destruction and no signs of primary infectious etiology. Anti-paraneoplastic antigen Ma2 (anti-PNMA2) IgG class autoantibodies were the only positive findings. Flow cytometry of CSF demonstrated a majority of lymphocytes (approximately 61%); most of the lymphocyte population was represented by T cells (approximately 95%), with the dominant proportion being CD4^+^ helper T cells (Fig. 4).

According to the recommendations for the management of irAE, high-dose intravenously administered corticosteroid therapy was started: Solu-Medrol (methylprednisolone) 2 mg/kg per day. Trimethoprim/sulfamethoxazole was administered simultaneously to prevent possible infectious complications: 960 mg twice a day (BID) twice a week. Despite the high dose of intravenously administered corticosteroid therapy, there was further deterioration of choreiform movements. The choreiform, athetoid, and ballistic movements spread to his lower limbs and trunk. The choreiform movements were so intense that our patient was unable to rest or lie on a bed. Furthermore, he developed a paranoid hallucinatory syndrome with suicidal thoughts. He was started on antipsychotic therapy (clonazepam 2 mg per day, haloperidol 15 mg per day, olanzapine 20 mg per day) after consultation with a psychiatrist and a neurologist and he experienced partial improvement. Considering the lack of a significant effect of corticosteroid therapy, the administration of infliximab at a dose of 5 mg/kg was started. However, infliximab did not achieve any clinical effect.

Our patient and his family insisted on discharge from our hospital. According to the conclusion of a psychiatric examination, he was pronounced capable of signing an against-medical-advice discharge form and was discharged on 23 August 2016. An early out-patient visit to administer a further dose of infliximab was scheduled, and he was duly informed about the importance of doing so. However, he did not come to visit and refused any further treatment despite the provided information about possible adverse consequences.

He was eventually admitted to the standard ward on 13 September 2016, presenting with a fever and soporific state (Glasgow Coma Scale 5) on admission. Because of urinary retention (initially 1000 ml) and elevated levels of C-reactive protein, we suspected urinary infection, and he was started on intravenously administered amoxicillin/clavulanic acid 3.6 g/day and intravenously administered hydration. No other laboratory abnormalities were found. He partially regained consciousness after 2 days of treatment, with clinical manifestations of aggression and choreiform movement as described above. Therefore, therapy with antipsychotics and corticosteroids was reintroduced. However, his condition started to deteriorate again, and he developed bronchopneumonia. His level of consciousness started to deteriorate again, and he died 4 days following admission.

**Fig. 5:**
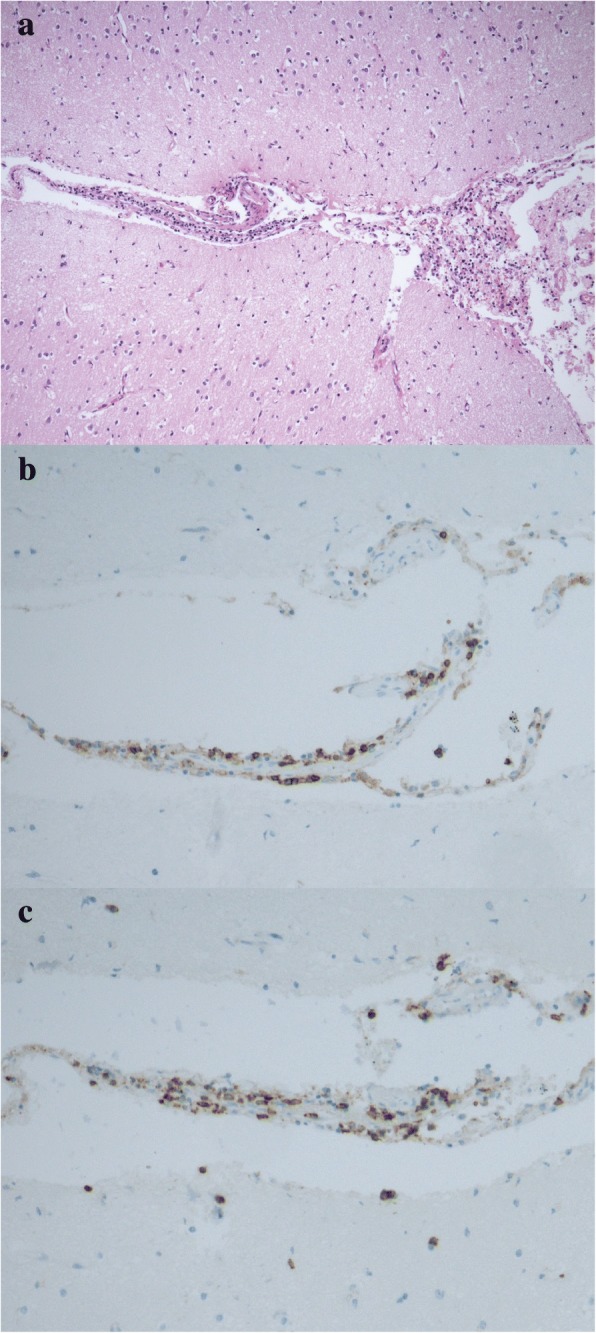
Histological samples of the brain (× 100 magnification). **a** Hematoxylin and eosin staining showing lymphocytic meningitis. **b** Immunohistochemical staining of affected brain tissue for CD4+ and **c** CD8+ markers

**Fig. 6:**
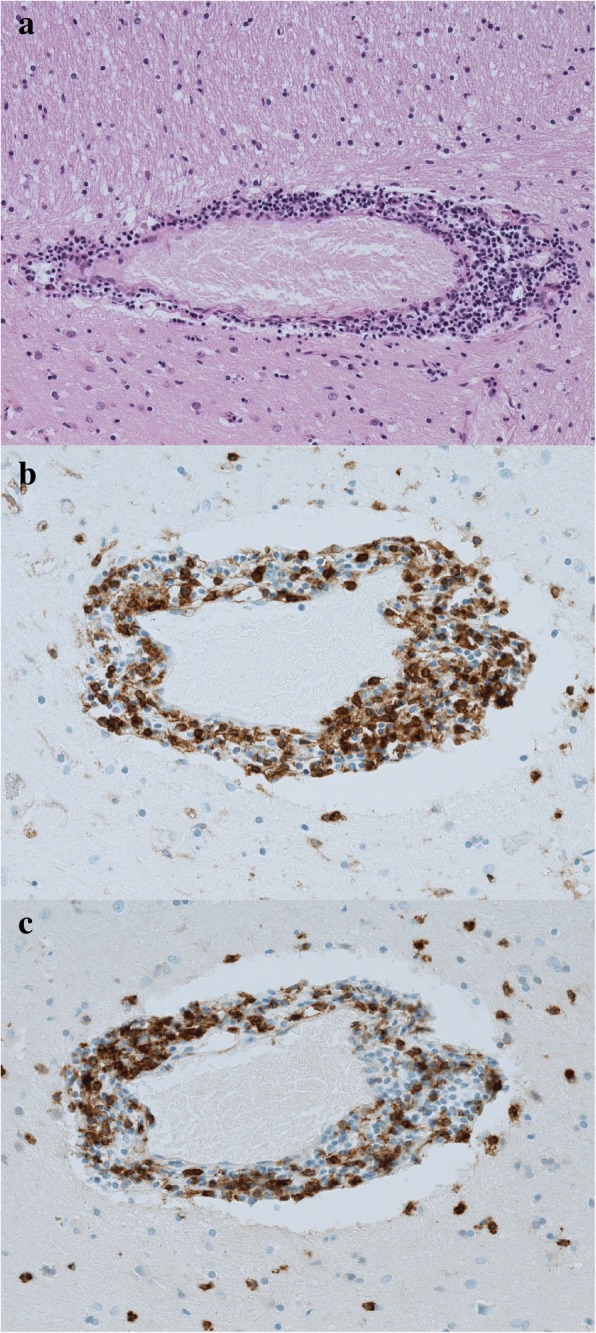
Histological samples of the basal ganglia (× 100 magnification). **a** Hematoxylin and eosin staining of perivascular infiltration. **b** Immunohistochemical staining of perivascular area of basal ganglia for CD4+ and **c** CD8+ markers

An autopsy confirmed the histology of clear cell renal cancer with metastatic para-aortic lymph nodes and necrotic Th11 vertebra, probably due to necrotic metastasis rather than radiotherapy-induced focal necrosis. Considering the clinical suspicion of aseptic meningitis, his brain was extensively examined. Its weight was 1480 g, and there were no macroscopically notable findings. A histological examination revealed inconclusive areas, suggesting focal lymphocytic meningitis of the entire brain—the cerebrum, brainstem, and cervical spinal cord (Fig. 5a)—and multiple perivascular lymphocytic infiltrates, which were most prominent in the basal ganglia on both sides; these findings were consistent with the MRI examination (Fig. 6a). The perivascular infiltrates localized in the frontal lobe and basal ganglia were immunohistochemically analyzed for surface markers of CD4 and CD8 T cells (Figs. 5b, c and 6b, c). The ratio of CD4^+^/CD8^+^, which is typically 3:1 in aseptic meningitis, was unusually low (approximately 1:1) in both sections.

## Discussion

Immunotherapy is a treatment modality that has experienced a renaissance in the treatment of solid tumors during the last decade. Despite numerous attempts to utilize the immune system to fight cancer, including the depletion of T-regulatory lymphocytes [2], and the use of cancer vaccines or cytokines [3], previous results have been unsatisfactory. However, the discovery and understanding of the function and regulatory role of several receptors and their ligands, that is, cytotoxic T-lymphocyte antigen-4 (CTLA-4), PD-1, and programmed death-ligand 1 (PD-L1) in T cell activation have encouraged the further development and clinical use of immunotherapy [4, 5]. Currently, we can affect these regulatory mechanisms with a new class of drugs called immune checkpoint inhibitors. There is a growing body of evidence that these drugs represent effective treatments with the possibility of achieving a durable response in a wide spectrum of solid tumors, including melanoma [6, 7], non-small cell lung cancer [8, 9], urothelial carcinoma [10, 11], and renal cell carcinoma (RCC) [1].

The pathogenesis of irAEs is closely related to immune regulation affecting the activity of cytotoxic T cells. The mechanism of action of checkpoint inhibitors is to disrupt an interaction between regulatory receptors and their ligands, which normally produce an inhibitory signal with subsequent attenuation of immune activity. If the binding of ligands to regulatory receptors is prevented, for example, by anti-PD-L1 or anti-PD-1 antibodies, the activated T cells can contribute to an antitumor response [12–14]. On the other hand, this leads to an imbalance and possible disruption of immunological tolerance, which might cause an uncontrolled immune response directed against self-tissue antigens. This may result in undesired inflammatory activity involving various organ systems, referred to as irAEs. The most commonly affected sites are the gastrointestinal tract, skin, and endocrine system, but any organ or system of the human body can be affected.

In our case the initial presentation of adverse symptoms related to nivolumab was poor but unusual with severe chorea-like dyskinesia symptoms. There is only sparse information in the literature about similar cases; that is why the diagnostic and therapeutic algorithm is more difficult than, for example, for immune-related pneumonitis. Our case shows and emphasizes the urgent need of up front resolute immunosuppressive therapy in cases of immune-related encephalitis. Based on our case we propose the paraneoplastic antigen Ma2 as a helpful biomarker in order to make a correct diagnosis and to distinguish immune-related encephalitis from other possible causes.

According to the literature, irAEs affecting the central nervous system (CNS) induced by anti-PD-1 antibody treatment are rare [15]. The vast majority of observed irAEs affecting the CNS have been related to anti-CTLA-4 antibodies [16, 17]. Most cases of immune checkpoint inhibitor-related CNS toxicities were of lower grade, with only approximately 0.1–3% of all events being grade 3 or 4 [18]. There are a limited number of anecdotal reports regarding anti-PD-1 antibody-induced autoimmune CNS toxicities [19–21].

Immune-mediated encephalitis can be difficult to distinguish from encephalitis related to other causes. It can produce a wide range of symptoms, including headache, fever, weakness, fatigue, impaired memory, hallucinations, and convulsions. The diagnosis is usually made *per exclusionem* (a diagnosis of exclusion)*.* Immune-mediated encephalitis induced by checkpoint inhibitors usually arises at the very beginning of therapy [15, 19]. However, as we demonstrate here, it can occur at any time during therapy.

The ratio of CD4^+^/CD8^+^ T cells in aseptic meningitis is typically approximately 3:1 [22]; however, it was unusually low in our case (approximately 1:1). This result is probably because the PD-1 receptor is strongly expressed on CD8^+^ T cells [23], resulting in the proliferation of the CD8^+^ subpopulation. Another explanation is that the CD4^+^/CD8^+^ ratio naturally decreases during inflammation, so the result might be affected by the time of examination [24].

The exact mechanism of immune-mediated encephalitis remains unclear. It is believed that the majority of irAEs are caused predominantly by T cells, although some proportion of irAEs may be mediated by other immune cells. N-methyl-D-aspartate (NMDA) receptor antibodies seem to play an important role in the pathogenesis of immune-mediated encephalitis [25]. These antibodies have been found in patients who developed immune-mediated encephalitis related to immune checkpoint inhibitor therapy [19]. NMDA receptors are expressed on the surface of melanocytes and are coded by the gene *GRIN2A*, which tends to be highly mutated in patients with melanoma [26]. The formation of antibodies against aberrant NMDA receptors in melanoma is supposed to induce encephalitis, as these antibodies may cross-react with NMDA receptors in the brain [15]. This mechanism has not been observed in mRCC yet. Antibodies against the NMDA receptor were not present in our patient, suggesting that other mechanisms are probably involved in the pathogenesis of checkpoint inhibitor-induced immune encephalitis.

Anti-Ma2-associated paraneoplastic neurological syndromes, such as brain encephalitis, usually present as isolated or combined limbic, diencephalic, or brainstem dysfunction [27, 28]. The presence of anti-Ma2 antibodies has been associated with neoplasms in approximately 96% of described cases [28], mainly cases of testicular cancer and small cell lung carcinoma. There is a growing body of evidence that anti-Ma2-associated encephalitis differs from typical paraneoplastic limbic or brainstem encephalitis and may therefore be unrecognized. The association of anti-Ma2 antibodies and mRCC has not been proven yet. However, the association between anti-Ma2 antibodies and immune encephalitis might be similar to that between anti-NMDA antibodies and melanoma. We cannot exclude the presence of anti-Ma2 antibodies prior to the start of checkpoint inhibitor therapy, but this finding undoubtedly contributed to the diagnosis of immune-mediated encephalitis in our patient.

The mainstay of the management of immune-mediated encephalitis is therapy with intravenously administered corticosteroids, similarly to that of other irAEs. However, therapy can be successful only if absolute compliance of the patient and his family can be guaranteed. The dose of corticosteroids depends on toxicity grade, ranging from 0.5 to 2 mg/kg per day [29]. The response to corticosteroid therapy in immune encephalitis seems to be slower and less pronounced than that in other irAEs. Therefore, higher initial doses of corticosteroids may be required. Alternatively, other immunosuppressive drugs such as infliximab can be used.

It must be emphasized that patients treated with intravenously administered corticosteroids are especially prone to infectious complications, and adequate care should be paid to this issue. The presented patient was administered trimethoprim/sulfamethoxazole as prevention against *Pneumocystis* pneumonia. Unfortunately, early signs of infection could not be recognized as he refused to remain in our hospital. It should therefore be recommended that patients with severe irAEs are treated in an in-patient setting in order to recognize early signs of infectious complications and start antibiotic treatment as soon as possible.

Unfortunately, we were not able to convince either our patient or his family to comply, so there was no way to extend our therapy (for example, with intravenously administered immunoglobulins or plasmapheresis) to prevent his death.

## Conclusions

The major problem with immune checkpoint inhibitors is the relatively short-term collective experience. Hence, there is an urgent need to extend physicians’ and patients’ knowledge of possible complications of this promising treatment modality. This is especially important for rare but potentially fatal complications. Therefore, it is of great importance to include this topic in multidisciplinary tumor board meetings. Considering the rarity of immune checkpoint inhibitor-related encephalitis, it is essential to report such cases and share experience regarding treatment approaches and diagnostic tools. Immune-related encephalitis is a severe and potentially fatal complication requiring immediate hospital admission and extensive immunosuppressive therapy. The examination of CSF for paraneoplastic antibodies such as anti-NMDA receptor and anti-Ma2 antibodies in order to distinguish autoimmune etiology from other possible causes is essential and highly recommended.

## Additional file

Additional file 1. Timeline of the case report. (PDF 177 kb)

## Abbreviations

anti-PNMA2: Anti-paraneoplastic antigen Ma2
BID: Twice a day
CNS: Central nervous system
CSF: Cerebrospinal fluid
CT: Computed tomography
CTLA-4: Cytotoxic T-lymphocyte antigen-4
Ig: Immunoglobuline
irAE: Immune-related adverse event
mRCC: Metastatic renal cell carcinoma
MRI: Magnetic resonance imaging
mTOR: Mammalian target of rapamycin
NMDA: N-methyl-D-aspartate
PD-1: Programmed cell death-1
PD-L1: Programmed death-ligand 1
RCC: Renal cell carcinoma
VEGF: Vascular endothelial growth factor
