# Supplementary material for: Nivolumab induced encephalopathy in a man with metastatic renal cell cancer: a case report
Source: J Med Case Rep. 2018 Sep 15;12:262. doi: 10.1186/s13256-018-1786-9 (PMC6138913; doi:10.1186/s13256-018-1786-9)
Supplement: Supplementary file 1 — Timeline of the case report. (PDF 177 kb) [file 13256_2018_1786_MOESM1_ESM.pdf]

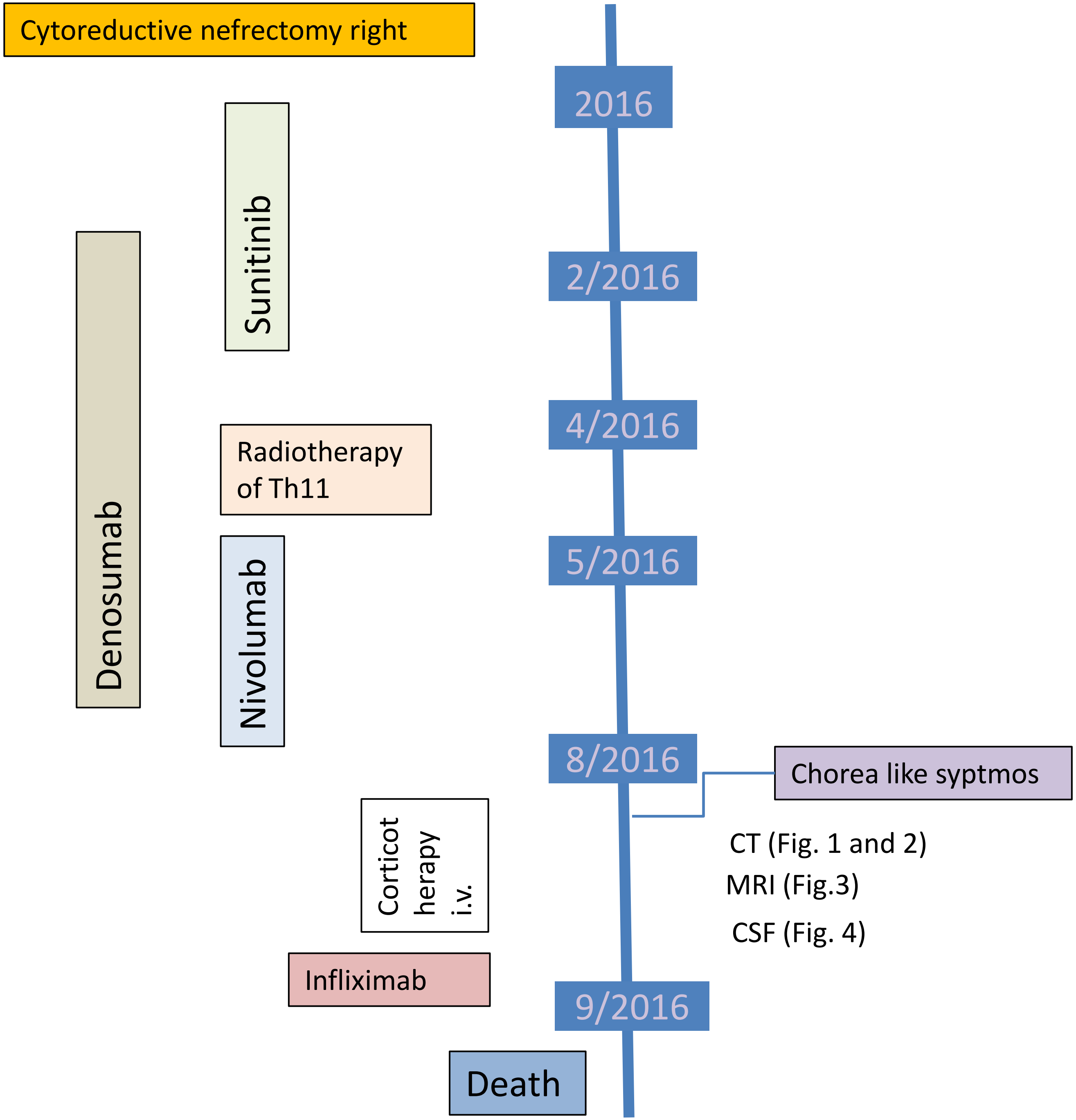
Cytoreductive nefrectomy right
2016
Sunitinib
Denosumab
2/2016
Radiotherapy of Th11
4/2016
Nivolumab
5/2016
8/2016
Chorea like syptmos
CT (Fig. 1 and 2)
MRI (Fig.3)
CSF (Fig. 4)
Corticot herapy i.v.
Infliximab
9/2016
Death
